# Validation of the Antidiabetic and Hypolipidemic Effects of *Clitocybe nuda* by Assessment of Glucose Transporter 4 and Gluconeogenesis and AMPK Phosphorylation in Streptozotocin-Induced Mice

**DOI:** 10.1155/2014/705636

**Published:** 2014-02-03

**Authors:** Chun-Ching Shih, Mei-Hsing Chen, Cheng-Hsiu Lin

**Affiliations:** ^1^Graduate Institute of Pharmaceutical Science and Technology, College of Health Science, Central Taiwan University of Science and Technology, No. 666 Buzih Road, Beitun District, Taichung City 40601, Taiwan; ^2^Plant Pathology Division, Taiwan Agricultural Research Institute, Council of Agriculture, Executive Yuan, Wufeng District, Taichung City 41362, Taiwan; ^3^Department of Internal Medicine, Fengyuan Hospital, Ministry of Health and Welfare, Fengyuan District, Taichung City 42055, Taiwan

## Abstract

The study was designed to investigate the effects of extract of *Clitocybe nuda* (CNE) on type 1 diabetes mellitus and dyslipidemia in streptozotocin- (STZ-) induced diabetic mice. Diabetes was induced by injection of STZ. Diabetic mice were randomly divided into five groups and given orally CNE (C1: 0.2, C2: 0.5, and C3: 1.0 g/kg body weight) or metformin (Metf) or vehicle for 4 weeks. STZ induction decreased in the levels of insulin, body weight, and the weight of skeletal muscle, whereas the levels of blood glucose, hemoglobin nonenzymatically (percent HbA1c), and circulating triglyceride (*P* < 0.001, *P* < 0.001, and *P* < 0.01, resp.) were increased. CNE decreased the levels of blood glucose, HbA1c, and triglyceride levels, whereas it increased the levels of insulin and leptin compared with the vehicle-treated STZ group. STZ induction caused a decrease in the protein contents of skeletal muscular and hepatic phosphorylation of AMP-activated protein kinase (phospho-AMPK) and muscular glucose transporter 4 (GLUT4). Muscular phospho-AMPK contents were increased in C2-, C3-, and Metf-treated groups. CNE and Metf significantly increased the muscular proteins of GLUT4. Liver phospho-AMPK showed an increase in all CNE- and Metf-treated groups combined with the decreased hepatic glucose production by decreasing phosphenolpyruvate carboxykinase (PEPCK), glucose-6-phosphatase (G6Pase), and 11beta hydroxysteroid dehydroxygenase (11*β*-HSD1) gene, which contributed to attenuating diabetic state. The study indicated that the hypoglycemic properties of CNE were related to both the increased muscular glucose uptake and the reduction in hepatic gluconeogenesis. CNE exerts hypolipidemic effect by increasing gene expressions of peroxisome proliferator-activated receptor *α* (PPAR*α*) and decreasing expressions of fatty acid synthesis, including acyl-coenzyme A: diacylglycerol acyltransferase (DGAT) 2. Therefore, amelioration of diabetic and dyslipidemic state by CNE in STZ-induced diabetic mice occurred by regulation of GLUT4, PEPCK, DGAT2, and AMPK phosphorylation.

## 1. Introduction

*Clitocybe nuda* (Fr.) Bigelow & Smith (*Lepista nuda,* commonly known as wood blewit or blue stalk mushroom) is an edible woodland mushroom found in Europe, North American, Asia, and Australia [1]. Due to its special fragrance and delicate texture, it has been cultivated in France, Holland, Britain, and Taiwan. Several bioactive extracts from *C. nuda* have been found to exhibit antioxidant and antimicrobial properties [2–4], but few reports have described medicinal activities and health benefit of human disorders. A new drimane sesquiterpenoid including 3-keto-drimenol, 3beta-hydroxydrimenol, and 3beta, 11, 12-trihydroxydrimene had been shown to exert inhibitory activities against two isozymes of 11beta-hydroxysteroid dehydrogenases (11*β*-HSD1), which catalyze the interconversion of active cortisol and inactive cortisone [5]. Inhibitors of 11*β*-HSD1 are known to have a potential treatment for the metabolic syndrome [6].

The prevalence of diabetes mellitus (DM) represents a chronic metabolic disorder and growing global health problem. The prevalence of diabetes is increasing globally and is predicted to raise by twofold from 150 million in the year 2000 to 300 million by the year 2030 [7]. Streptozotocin (STZ), a nitrosurea derivative, is one of the most universally used diabetogenic agents to induce diabetes in experimental animals [8]. It is prominent for its selective pancreatic *β*-cell cytotoxicity and has been extensively used to induce insulin-dependent diabetes mellitus or type I diabetes [9]. STZ is taken up by pancreatic *β* cells and subsequently induces their death. Collectively, the potent alkylating properties of STZ are the main reason for its toxicity. STZ is a nitric oxide donor, nitric oxide was found to bring about the destruction of pancreatic islet cells, and STZ by itself was found to generate reactive oxygen species, which contributed to DNA fragmentation and evoked other deleterious changes in the cells. Therefore, the synergistic action of both nitric oxide and reactive oxygen species may also contribute to DNA fragmentation and other deleterious changes [10, 11].

Glucose transporter 4 (GLUT4) is the major insulin-regulated glucose transporter expressed mainly in skeletal muscle and adipose tissue [12, 13]. Insulin stimulates glucose uptake in these cells primarily by inducing net translocation of GLUT4 from the intracellular storage sites to the plasma membrane [14]. Impairment of GLUT4 expression, GLUT4 translocation, and/or insulin signaling may affect insulin-stimulated glucose uptake, and that would result in insulin resistance and hyperglycemia [15, 16]. Therefore, the improvements of GLUT4 contents and/or translocation to the plasma membrane have long been regarded as a potential target in the treatment of diabetes mellitus. GLUT4 translocation is mainly regulated by two pathways: the insulin signaling pathway and AMP-activated protein kinase (AMPK) pathway [17].

The stress kinase, AMPK, has also been shown to regulate GLUT4 translocation [17], and therefore we investigated whether CNE activated AMPK in liver tissue and skeletal muscle. However, the antidiabetic activity of CNE is not well defined in streptozotocin-induced diabetic mice. AMPK is considered as a therapeutic target for treatment of diabetes and dyslipidemia [18, 19]. Activation of the AMPK results in increased lipid and glucose catabolism [20]. Phosphorylation of Thr 172 of *α* subunits is essential for AMPK activity [21]. As one of the possible mechanisms of action, this study also examined the effect of *Clitocybe nuda* (CNE) on the expression of genes involved in antidiabetes, lipogenesis, and triglyceride lipase in the liver tissue, including phosphoenolpyruvate caboxykinase (PEPCK), glucose 6-phosphatase (G-6Pase), 11beta-HSD1, and diacylglycerol acyltransferase 2 (DGAT2).

## 2. Materials and Methods

### 2.1. Materials and Preparation of Extract of *Clitocybe nuda* (CNE)

The mushroom (or fungal) strain of *Clitocybe nuda* (stain Tainung number 1) was cultured under compost extract agar medium. The preparation of grain spawn was as follows: wheat grains were washed with distilled water, boiled for 20 min, and removed from water by filtration. Then, they were added to 1% CaCO_3_ and mixed well, transferred to the flask, and were sterilized at 121°C and 1.2 kg/cm^3^ for 1 h. After one day, the hyphal chunk of the described above *C. nuda* was implanted to the flask at 24°C for 15 days for mycelia to cover grains, called grain spawn. The fruiting of *C. nuda* is as follows: the grain spawn of *C. nuda* was mixed with the fermented rice straw compost, incubated at 24°C for 21–28 days for spawn running, and covered with 1-2 cm peat and the condition was 13°C, 90–95% relative humidity, 1000 ppm CO_2_ concentration, and at daylight for 8 h. During the period, they were periodically supplied with water until fruiting, and then the mushrooms were harvested. After lyophilizing, 30 g of the dried mushroom samples was homogenized and extracted with 40 times volumes of hot water under reflux at 100°C for 40 min. The aqueous extract was filtered over Whatman number 1 paper and the filtrate was evaporated to a small volume. The filtrate was lyophilized and designated the hot-water soluble fraction (CNE) and was stored frozen at −20°C until required. The total phenolic contents were determined by the Folin-Ciocalteau method [22]. The total phenolic contents of CNE were 1.29%. The polysaccharides of CNE were 12.62% by phenol-sulfuric acid method [23]. The total anthocyanin contents of CNE were 0.045%. The CNE was diluted adjusted, and then administrated orally to mice in a volume of 0.2, 0.5, and 1.0 g/kg bodyweight (C1: 0.2, C2: 0.5, and C3: 1.0 g/kg bodyweight), respectively. Distilled water was administered in a similar volume to control mice.

### 2.2. Acute Effects of CNE

The acute effects of CNE on fasting glucose were studied in diabetic mice. Animals were fasted for 15–18 h but were allowed access to water. Initial blood samples were obtained for determination of basal glucose levels from retroorbital sinus using heparinized capillary tubes. Blood samples were then taken at 1, 3, 5, and 7 h following dosing with 0.5 and 1.0 g/kg CNE or with an equivalent volume of normal saline.

### 2.3. Experimental Chronic Induction of Diabetic Mice

Male C57BL/6J mice, aged 5 weeks, were used. Diabetes was induced by intraperitoneal injection of streptozotocin (Sigma Chemical, St. Louis, MO, USA) for five consecutive days. The dosage of STZ is 55 mg/kg (dissolved in 0.05 M cold sodium citrate buffer, pH 4.5). The normal control mice received only citrate buffer of the same volume. After 2 weeks, the mice with severe diabetes exerting hyperglycemia (fasting blood glucose range of above 250 mg/dL) were considered as diabetic and selected for the experiment. Diabetic mice were randomly divided into five groups and were either treated with vehicle (distilled water), CNE (0.2, 0.5, or 1.0 g/kg), or metformin (300 mg/kg) in a similar volume. The vehicle, CNE, or metformin was administered orally to mice once daily and thereafter for 28 days. During the experiment, all mice were fasted overnight and blood was collected from retroorbital sinus under ether anaesthesia. At the end of the experiment, mice were sacrificed by carbon dioxide inhalation. Liver, adipose tissue, and skeletal muscle were removed and immediately frozen in liquid nitrogen and stored at −80°C for various assays. Blood sample was allowed to clot at 25°C for 5 min. Plasma samples were collected by centrifugation at 1600 ×g for 15 min at 4°C. The separation of the plasma was finished within 30 min. Aliquots of the supernatant were obtained for total cholesterol (TC) and triglyceride (TG) assay and immediately frozen at −80°C until use.

### 2.4. Analysis of Fasting Blood Glucose and Biochemical Parameters

Blood samples were collected from the retroorbital sinus of fasting mice and the glucose level was measured by the glucose oxidase method (Model 1500; Sidekick Glucose Analyzer; YSI Incorporated, Yellow Springs, USA). The concentrations of TG and TC were measured using commercial assay kits according to the manufacturer's directions (Triglycerides-E test and Cholesterol-E test, Wako Pure Chemical, Osaka, Japan).

### 2.5. Analysis of Adipocytokine Levels

The levels of insulin and leptin were measured by ELISA using a commercial assay kit according to the manufacturer's directions (mouse insulin ELISA kit, Sibayagi, Gunma, Japan, and mouse leptin ELISA kit, Morinaga, Yokohama, Japan).

### 2.6. Histology Analysis of Epididymal WAT and Liver Tissue

Small pieces of epididymal WAT and liver were fixed with formalin (200 g/kg) neutral buffered solution and embedded in paraffin. Sections (8 *μ*m) were cut and stained with hematoxylin and eosin. For microscopic examination, a microscope (Leica, DM2500) was used, and the images were taken using a Leica Digital camera (DFC-425-C) at 10 (ocular) × 20 (object lens) magnification.

### 2.7. Isolation of RNA and Relative Quantization of mRNA Indicating Gene Expression

Total RNA from the epididymal WAT and liver was isolated with a Trizol Reagent (Molecular Research Center, Inc., Cincinnati, OH, USA) according to the manufacturer's directions. The integrity of the extracted total RNA was examined by 2% agarose gel electrophoresis, and the RNA concentration was determined by the ultraviolet (UV) light absorbency at 260 nm and 280 nm (Spectrophotometer U-2800A, Hitachi). The quality of the RNA was confirmed by ethidium bromide staining of 18S and 28S ribosomal RNA after electrophoresis on 2% agarose gel containing 6% formaldehyde. Total RNA (1 *μ*g) was reverse transcribed to cDNA in a reaction mixture containing buffer, 2.5 mM dNTP (Gibco-BRL, Grand Island, NY, USA), 1 mM of the oligo (dT) primer, 50 mM dithiothreitol, 40 U Rnase inhibitor (Gibco-BRL, Grand Island, NY, USA), and 5 *μ*L Moloney murine leukemia virus reverse transcriptase (Epicentre, USA) at 37°C for 1 h and then heated at 90°C for 5 min to terminate the reaction. The polymerase chain reaction (PCR) was performed in a final 25 *μ*L containing 1 U Blend Taq-Plus (TOYOBO, Osaka, Japan), 1 *μ*L of the RT first-strand cDNA product, 10 *μ*M of each forward (F) and reverse (R) primer, and 75 mM Tris-HCl (pH 8.3) containing 1 mg/L Tween 20, 2.5 mM dNTP, and 2 mM MgCl_2_. Preliminary experiments were carried out with various cycles to determine the nonsaturating conditions of the PCR amplification for all the genes studied. The primers are shown in Tables 1 and 2. The products were run on 2% agarose gels and stained with ethidium bromide. The relative density of the band was evaluated using AlphaDigiDoc 1201 software (Alpha Innotech, Co., San Leandro, CA, USA). All the measured PCR products were normalized to the amount of cDNA of GAPDH in each sample.

### 2.8. Western Immunoblotting Analysis of Phospho-AMPK (Thr172) Proteins

Protein extractions and immunoblots for the determination of AMPK phosphorylation were carried out on frozen liver tissue and skeletal muscle from mice according to a previous report [24]. Briefly, liver samples (0.1 g) were powdered under liquid nitrogen and homogenized for 20 s in 500 *μ*L buffer containing 20 mM Tris-HCl (pH 7.4 at 4°C), 2% SDS, 5 mM EDTA, 5 mM EGTA, 1 mM DTT, 100 mM NaF, 2 mM sodium vanadate, 0.5 mM phenylmethylsulfonyl fluoride, 10 *μ*g/mL leupeptin, and 10 *μ*L/mL pepstatin [25]. 40 *μ*g of each homogenate was mixed with an equal amount of 2 × standard SDS sample loading buffer containing 125 mM Tris-HCl (pH 6.8), 4% SDS, 20% glycerol, 10% *β*-mercaptoethanol, and 0.25% bromophenol blue and boiled for 10 min before electrophoresis. Proteins were separated by 12% SDS-PAGE according to the method of Laemmli [26] and transferred by electroblotting onto PolyScreen PVDF transfer membrane (NEN) using semidry transfer cell (Bio-Rad) according to the manufacturer's manual. The membrane was then treated sequentially with blocking solution (phosphate-buffered saline (PBS) containing 5% nonfat skim milk), with appropriate dilution of anti-phospho-AMPK*α* (Thr 172) antibody (Abcam Inc., Cambridge, MA, USA) and with anti- G6PD (G6PD) (glucose 6 phosphate dehydrogenase antibody; Abcam Inc, USA) conjugated to peroxidase (Zymed Inc., South San Francisco, CA, USA). Finally, the membrane was soaked in a chromogen/substrate solution (TMB single solution; Zymed) for color development.

### 2.9. Statistical Analysis

Data were expressed as mean ± S.E. values. Whenever possible, data were subjected to analysis of variance, followed by Dunnett's multiple range test, using SPSS software (SPSS Inc., Chicago, IL, USA). *P* < 0.05 was considered to be statistically significant.

## 3. Results

### 3.1. Acute STZ Induced Diabetic Mice

**Figure 1:**
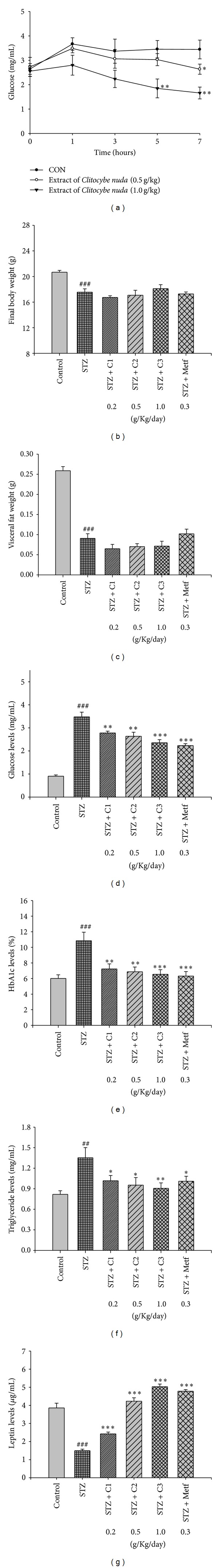
Effects of extract of *Clitocybe nuda* on (a) the levels of blood glucose in STZ induced acute test, (b) final body weight, (c) visceral fat weight, (d) blood glucose levels, (e) the glycosylated hemoglobin (HbA1c) levels, (f) circulating triglyceride levels, and (g) leptin levels at the end of experiment in STZ induced chronic test. All values are means ± S.E. (*n* = 9). ^#^ *P *< 0.05, ^##^ *P *< 0.01, and ^###^ *P *< 0.001 compared with the control (CON) group; *P < 0.05, **P < 0.01, and ***P < 0.001 compared with the streptozotocin + vehicle (distilled water) (STZ) group. C1, C2, and C3: extracts of *Clitocybe nuda* (C1: 0.2, C2: 0.5, and C3: 1.0 g/kg bodyweight); Metf: metformin (0.3 g/kg body weight). The HbA1c is represented by % hemoglobin.

Following treatment with 0.5 g/kg CNE, blood glucose levels were decreased after 7 h. In the treatment with 1.0 g/kg CNE, blood glucose levels were decreased after 5 h and the hypoglycemic effect continued to 7 h (Figure 1(a)).

### 3.2. Chronic Effects

#### 3.2.1. Body Weight and Tissue Weight

All group mice started with similar mean body weights at the beginning of the study (19.6 ± 0.4 g). At the end of the experiment, the STZ induced mice caused a significant decrease in body weight and skeletal muscle weight compared with the CON group (*P* < 0.001, *P* < 0.001, resp.). There is no significant difference in the body weight between the CNE- and Metf-treated STZ group and vehicle-treated STZ group (Figure 1(b)). STZ induction caused a decrease in the weight of white adipose tissue (WAT) (including epididymal, mesenteric WAT, and visceral fat) (*P* < 0.001, *P* < 0.001, and *P* < 0.001, resp.) and skeletal muscle (*P* < 0.001). There is no significant difference in the weights of retroperitoneal WAT, visceral fat, and skeletal muscle between the CNE-treated STZ group and vehicle-treated STZ group (Figure 1(c) and Table 2).

#### 3.2.2. Plasma Glucose Levels and Blood Glycated Hemoglobin (HbA1c)

At the beginning of the study, all mice started with similar levels. At the end of the experiment, the glucose levels of the STZ group were significantly greater than the CON group (*P* < 0.001). Treatment with C1, C2, C3, and Metf showed a significant reduction in plasma glucose compared with the vehicle-treated STZ group (*P* < 0.01, *P* < 0.01, *P* < 0.001, and *P* < 0.001, resp.) (Figure 1(d)). The percent of hemoglobin was evaluated nonenzymatically (percent HbA1c), as an integrated measure of long-term blood glucose regulation. STZ induction caused an increase in HbA1c in the STZ group compared to the CON group (*P* < 0.001). After treatment, all CNE- and Metf-treated groups showed a significant reduction in HbA1c as compared with the STZ group (Figure 1(e)).

#### 3.2.3. Plasma Lipid

At the end of the experiment, the levels of TC and TG were 48.1% and 65.4% greater in the STZ group than in the CON group (*P* < 0.001, *P* < 0.01, resp.). Treatment with C1, C2, C3, and Metf suppressed the STZ diet-induced increases in the concentrations of TG (*P* < 0.05, *P* < 0.05, *P* < 0.05, and* P* < 0.05, resp.). There is no significant difference in the concentrations of TC between the CNE-treated STZ group and vehicle-treated STZ group (Table 2 and Figure 1(f)).

#### 3.2.4. Insulin and Leptin Concentration

As shown in Table 2 and Figure 1(g), at the end of the experiment, the concentrations of insulin and leptin were lower in the STZ group than in the CON group (*P *< 0.001, *P *< 0.001, resp.). C1, C2-, C3,- and Metf-treated groups significantly increased the levels of insulin compared with the vehicle-treated STZ group (*P* < 0.01, *P* < 0.01, *P* < 0.001, and *P* < 0.01, resp.). C1-, C2-, C3-, and Metf-treated groups significantly increased leptin levels compared with the vehicle-treated STZ group (*P* < 0.01, *P* < 0.001, *P* < 0.001, and* P* < 0.001, resp.).

#### 3.2.5. Histology of Epididymal WAT and Liver Tissue

**Figure 2:**
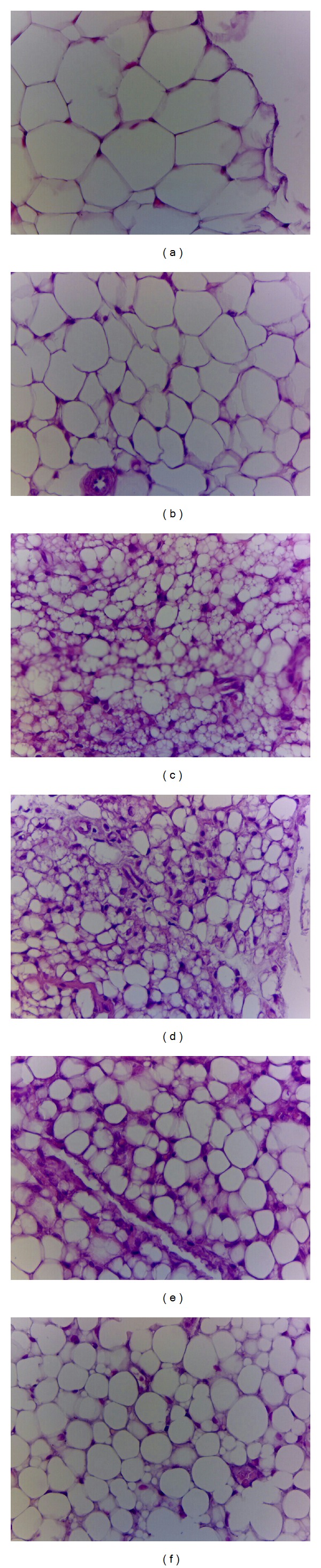
Histology of epididymal white adipose tissue (WAT) of mice in the (a) control (CON), (b) streptozotocin + vehicle (distilled water) (STZ), (c) STZ + C1, (d) STZ + C2, (e) STZ + C3, or (f) STZ + metformin (Metf) groups by hematoxylin and eosinstain. Magnification: 10 (ocular) × 20 (object lens). C1, C2, and C3: extracts of *Clitocybe nuda* (C1: 0.2, C2: 0.5, and C3: 1.0 g/kg bodyweight); Metf: metformin (0.3 g/kg body weight).

**Figure 3:**
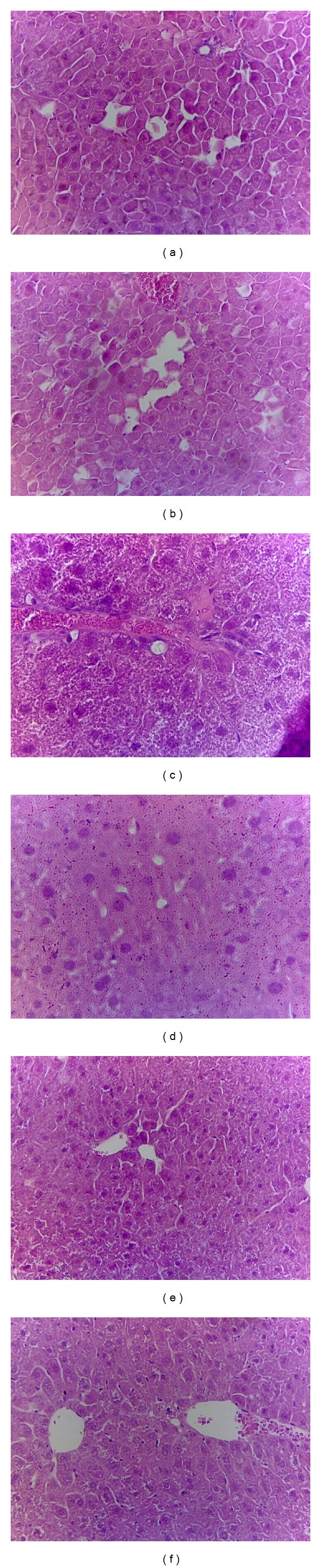
Histology of liver tissue of mice in the (a) control (CON), (b) streptozotocin + vehicle (distilled water) (STZ), (c) STZ + C1, (d) STZ + C2, (e) STZ + C3, or (f) STZ + metformin (Metf) groups by hematoxylin and eosinstain. Magnification: 10 (ocular) × 20 (object lens). Mice treated with the streptozotocin + vehicle (distilled water) (STZ) group. C1, C2, and C3: extracts of *Clitocybe nuda* (C1: 0.2, C2: 0.5, and C3: 1.0 g/kg bodyweight); Metf: metformin (0.3 g/kg body weight).

STZ induced the adipocytes smaller than the CON group in epididymal WAT. Following treatment with C1 and C2 caused the atrophy compared with the vehicle-treated STZ group. The results obtained from the other mice are similar to those shown in Figure 2. STZ induction does not cause ballooning of hepatocyte compared with the CON group. Afterwards, treatment with C1, C2, C3, and Metf exerts no ballooning phenomenon. These morphological results strongly suggest that neither STZ induction nor CNE caused the hepatic TG accumulation. The results obtained from the other mice are similar to those shown in Figure 3.

#### 3.2.6. Expressions of PEPCK, 11*β*-HSD1, Glucose-6-Phosphatase (G-6Pase), Peroxisome Proliferator-Activated Receptor *α* (PPAR*α*), Adipose Triglyceride Lipase (ATGL), and DGAT2 in Liver Tissue

**Figure 4:**
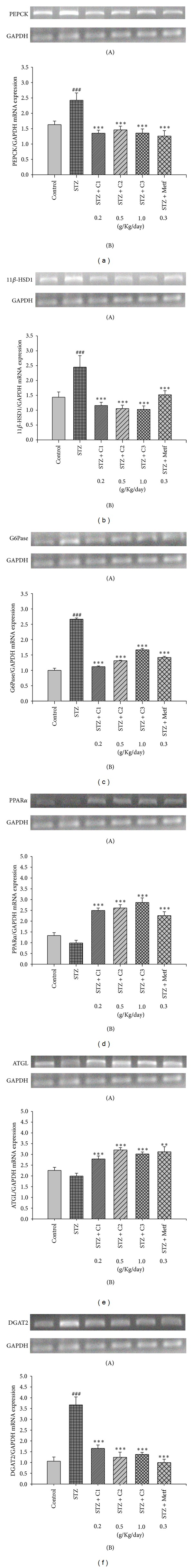
Semiquantitative RT-PCR analysis on (a) PEPCK, (b) 11*β*-HSD1, (c) G6Pase, (d) PPAR*α*, (e) ATGL, and (f) DGAT2 mRNA expression in liver tissue of the mice by oral gavage extracts of *Clitocybe nuda* (C1: 0.2, C2: 0.5, and C3: 1.0 g/kg bodyweight); Metf: metformin (0.3 g/kg body weight). Total RNA (1 *μ*g) isolated from tissue was reverse transcripted by MMLV-RT; 10 *μ*L RT products were used as templates for PCR. The expression levels of PEPCK, 11*β*-HSD1, G6Pase, PPAR*α*, ATGL, and DGAT2 mRNA were measured and quantified by image analysis. Values were normalized to GAPDH mRNA expression. All values are means ± S.E. (*n* = 9). ^#^ *P* < 0.05, ^##^ *P* < 0.01, and ^###^ *P* < 0.001 compared with the control (CON) group; *P < 0.05, **P < 0.01, and ***P < 0.001 compared with the streptozotocin + vehicle (distilled water) (STZ) group.

As shown in Figure 4, at the end of the experiment, the mRNA levels of PEPCK, 11*β*-HSD1, glucose-6-phosphatase (G6Pase), and DGAT2 were higher in the STZ group than in the CON group, whereas there was no significant difference in PPAR*α* and ATGL expression of mRNA in the STZ group compared with the CON group. Following treatment, the C1-, C2-, C3-, and Metf-treated groups significantly decreased the mRNA level of PEPCK (*P* < 0.001, *P* < 0.001, *P* < 0.001, and *P* < 0.001, resp.), 11*β*-HSD1 (*P* < 0.001, *P* < 0.001, *P* < 0.001, and *P* < 0.001, resp.), G6Pase (*P* < 0.001, *P* < 0.001, *P* < 0.001, and *P* < 0.001, resp.), and DGAT2 (*P* < 0.001, *P* < 0.001, *P* < 0.001, and *P* < 0.001, resp.). Following treatment, the C1-, C2-, C3-, and Metf-treated groups significantly increased the mRNA level of PPAR*α* (*P* < 0.001, *P* < 0.001, *P* < 0.001, and *P* < 0.001, resp.) and ATGL (*P* < 0.001, *P* < 0.001, *P* < 0.001, and *P* < 0.01, resp.) as compared with vehicle-treated STZ group (Figure 4).

#### 3.2.7. The Phospho-AMPK (Thr172) Protein Contents in Liver Tissue and Skeletal Muscle

**Figure 5:**
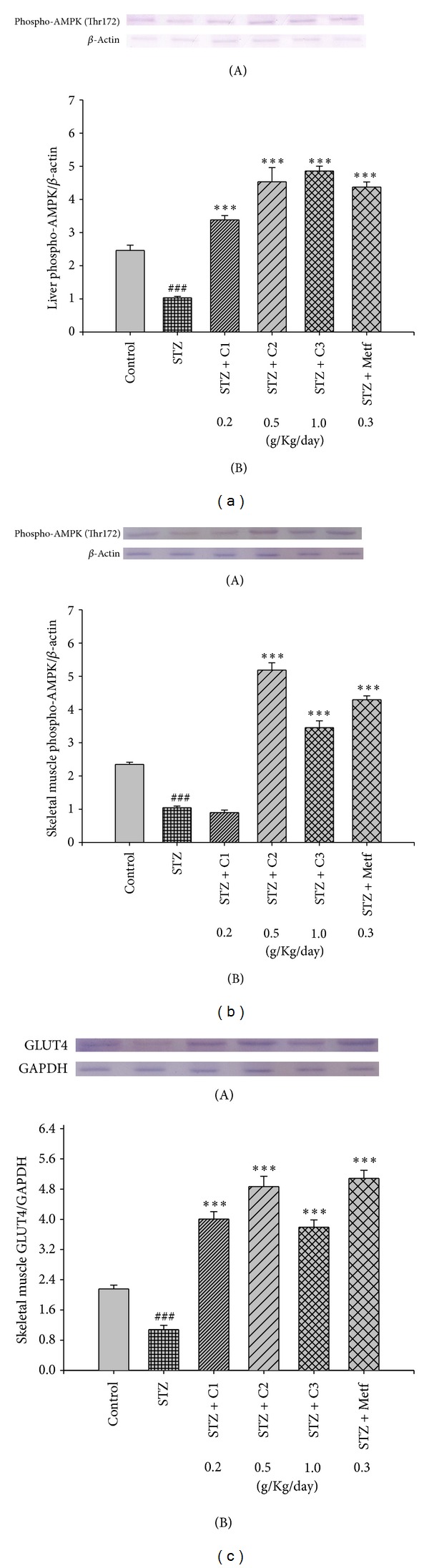
The phospho-AMPK (Thr172) protein contents in (a) liver and (b) skeletal muscle and (c) GLUT4 protein contents inskeletal muscle of the mice by oral gavage extract of *Clitocybe nuda*. Protein was separated by 12% SDS-PAGE detected by western blot. All values are means ± S.E. (*n* = 9). ^#^ *P* < 0.05, ^##^ *P* < 0.01, and ^###^ *P* < 0.001 compared with the control (CON) group; *P < 0.05, **P < 0.01, and ***P < 0.001 compared with the streptozotocin + vehicle (distilled water) (STZ) group. C1, C2, and C3: extracts of *Clitocybe nuda* (C1: 0.2, C2: 0.5, and C3: 1.0 g/kg bodyweight); Metf: metformin (0.3 g/kg body weight).

At the end of the experiment, the protein contents of phospho-AMPK protein were lower in the STZ group than in the CON group in liver and skeletal muscle (*P* < 0.001, *P* < 0.001, resp.). After treatment, the protein contents of hepatic phospho-AMPK were increased in the C1-, C2-, C3-, and Metf-treated groups compared with the STZ group (*P* < 0.001, *P* < 0.001, *P* < 0.001, and *P* < 0.001, resp.) (Figure 5(a)). Following treatment, the muscular protein contents of phospho-AMPK were increased in the C2, C3, and Metf-treated groups compared with the STZ group (*P* < 0.001, *P* < 0.001, and *P* < 0.001, resp.) (Figure 5(b)).

#### 3.2.8. The GLUT4 Protein Contents in Skeletal Muscle

At the end of the experiment, the protein contents of GLUT4 protein were lower in the STZ group than in the CON group in skeletal muscle (*P* < 0.001). After treatment, the skeletal muscular protein contents of GLUT4 were greater in C1, C2, C3-, and Metf-treated groups than in the STZ group (*P* < 0.001, *P* < 0.001, *P* < 0.001, and *P* < 0.001, resp.) (Figure 5(c)).

## 4. Discussion

The primary aim of the present study was to examine the effects and mechanism of CNE-mediated glucose lowering effect in a diabetic model, STZ induced diabetic mice, and to compare with metformin. STZ induced diabetic models have been used as type 1 diabetic models [27]. STZ is pancreatic *β*-cell toxin that induces rapid and irreversible necrosis of *β* cells. STZ induced model had also elevated levels of triglycerides. Therefore, STZ mouse model was chosen to address antidiabetic and hypolipidemic properties of CNE.

The present study demonstrated that the CNE exerted antihyperglycemic effect both in acute and chronic STZ induced diabetic mice. Our result that metformin reduced blood glucose in diabetes is consistent with results from a previous study [28]. To examine the antidiabetic properties of CNE, we chose GLUT4 translocation in skeletal muscle as the target. This value measures the movement of the insulin responsive glucose transporter GLUT4 to the cell surface, an essential step for insulin-responsive glucose in skeletal muscle. GLUT4 translocation of skeletal muscle was reduced in STZ induced diabetic rats [29]. Treatment with CNE exerted a significant increase in GLUT4 protein contents and exhibits a strong effect to stimulate GLUT4 translocation by severalfold in skeletal muscle to a level that was comparable to metformin stimulation, suggesting that the hypoglycemic effect of CNE was related to glucose uptake by skeletal muscle.

Hypertriglyceridaemia and hypercholesterolaemia have been reported to occur in STZ diabetic animals [30, 31]. Our findings of STZ induction may elevate levels of triglycerides and total cholesterol is in agreement with the results of Choi et al. [30] and Sharma et al. [31]. The data presented show that repeated treatment with CNE decreased plasma levels of triglycerides in diabetic mice. Thus, it was clearly shown that CNE had an improving effect on the hypertriglycemia induced by STZ. Nevertheless, treatment with CNE did not decrease the levels of total cholesterol in STZ induced mice.

Glycosylated hemoglobin is a biochemical marker that strongly correlates with the level of ambient glycemia during a 2- to 3-month period and is a more accurate and reliable measure than fasting blood glucose level [32]. Glycosylated hemoglobin level is known as a key target for the prognosis of diabetes-related complications [33]. The observed increase in the level of glycosylated hemoglobin in the experimental diabetic mice implies the oxidation of sugars and extensive damage to both sugars and proteins in some tissues, continuing and reinforcing the cycle of oxidative stress and damage [34]. Treatment with CNE significantly decreased the concentrations of glycosylated hemoglobin, suggesting that it may prevent oxidative damage caused by the glycation reaction in diabetic states. The present results of levels of glucose and glycosylated hemoglobin demonstrate that CNE exerts the beneficial effects in preventing the pathogenesis of diabetic complications caused by impaired glucose metabolism.

Streptozotocin-induced diabetes is characterized by a severe loss in body weight [35]. The decrease in body weight is due to both the loss and degradation of structural proteins [36] and the altered carbohydrate metabolism [37]. Insulin is an important anabolic hormone. Due to decreased production of ATP and absolute or relative deficiency of insulin, protein synthesis was decreased in all tissues [38]. In the present study, the results of STZ induction decreased body weight and insulin levels were in accordance with a previous study. Following treatment with CNE, there was no difference in body weights. CNE caused a significant increase in insulin levels and decrease in blood glucose levels. Our study demonstrated that CNE exerts antidiabetic activity. The ability of CNE to reduce blood glucose levels in diabetic mice is due to its potential of secreting insulin from the existing islet *β* cells.

GLUT4 is the rate-limiting step for glucose uptake in skeletal muscle. Skeletal muscle is the major tissue responsible for insulin-mediated glucose utilization [39]. Insulin stimulates glucose uptake in skeletal muscle by promoting the translocation of the GLUT4 to the plasma membrane [40]. GLUT4 proteins of skeletal muscle were reduced in STZ induced diabetic animals [41]. In this study, there was a significant increase in the protein contents of GLUT4 in the CNE-treated diabetic groups. Increased protein contents of GLUT4 demonstrated that CNE improved glucose utilization in skeletal muscle by restoring translocation of GLUT4 to the plasma membrane.

The liver plays a vital role in carbohydrate metabolism. Hepatic glucose overproduction is a crucial factor in diabetic hyperglycemia. Hepatic gluconeogenesis accounts for approximately 60%~97% of the hepatic glucose production [42]. PEPCK is a key rate-limiting enzyme of gluconeogenesis. The activities of glucose 6-phosphatase (G-6Pase) increased significantly in the liver of diabetic rats [43]. G-6Pase plays a role in glucose homeostasis [44]. Insulin integrates hepatic carbohydrate metabolism by increasing the biosynthesis of enzymes of glycolysis and glycogenesis and by inhibiting gluconeogenesis [45]. Treatment with CNE reduced the expressions of these enzymes including PEPCCK and G-6Pase. Furthermore, selective inhibition of 11*β*-HSD1 has been shown to improve hepatic insulin sensitivity in hyperglycemic KKAy mice [46]. Thus, compounds that decrease 11*β*-HSD1 may impart antidiabetic effects and promote insulin sensitivity. In this study, 11*β*-HSD1 mRNA was decreased in all CNE-treated groups in liver tissue. Therefore, in addition to downregulation of PEPCK and G-6Pase, a decrease of 11*β*-HSD1 also contributes to the antidiabetic effect of CNE. The increased insulin and decreased glucose in STZ-diabetic mice might be the results of restoration of these carbohydrate metabolism enzymes.

The phosphorylation of AMPK pathway is another major regulator of GLUT4 translocation during exercise or in response to some antidiabetic agents such as AICAR and metformin [17]. We found that C2 and C3 were able to increase the phosphorylation of AMPK in muscle to a level comparable to metformin. Whether the phosphorylation of AMPK is responsible for the stimulation of GLUT4 translocation by the CNE remains to be further studied.

Metformin acts by increasing the phosphorylation and activation of AMPK [47]. Activation of AMPK is known to decrease hepatic glucose production, and the overall effect is to decrease glucose levels [47]. Liver phospho-AMPK proteins were increased in CNE- and Metf-treated groups. This might also indicate that CNE has the ability to improve hyperglycemia through AMPK activities in gluconeogenesis. Therefore, it is possible that CNE caused glucose lowering both by activation of AMPK, and inhibiting hepatic glucose production via PEPCK and G-6Pase downregulation.

The second objective of this study was to look into the antihyperlipidemic effect of CNE. PPAR*α* is highly expressed in liver and controls *β*-oxidation [48]. To evaluate whether the effects of CNE on lipid profiles were mediated by alterations in PPAR*α* target gene expression in liver, we measured the mRNA levels of various targets, including DGAT2. PPAR*α* ligands are used widely to lower triglyceride levels in dyslipidemia and coronary heart disease [48]. Adipose triglyceride lipase (ATGL) is responsible for triacylglycerol hydrolase activity in cells that control the rate-limiting step of lipolysis in many insulin sensitive tissues. ATGL has been considered as a possible therapeutic target for dyslipidemia and fatty liver [49]. In this study, STZ induced diabetic mice exert lower PPAR*α* mRNA levels than the CON mice. Our findings show that STZ induced mice fed with CNE were found to have significantly higher ATGL and PPAR*α*, and lower DGAT2 mRNA levels than vehicle-treated STZ mice. However, CNE administration substantially enhanced hepatic PPAR*α* functions in STZ induced mice. In this study, following treatment with CNE, triglycerides lowering occurred as a result of downregulation of the enzyme, DGAT2, which catalyzes the final step in the synthesis of triglycerides [50]. Therefore, the downregulation of DGAT2 appears to be responsible for the hepatic triglyceride output, which, in turn, contributed to the lowering of circulating triglycerides. In this study, CNE caused decrease in serum levels of triglycerides and these further confirm CNE's lipid-lowering effects via downregulation of genes involved in lipid synthesis.

Another finding of this study showed that the treatment of mice with CNE significantly increased leptin concentrations. Minokoshi et al. [51] demonstrated that leptin activated AMPK. The activation is strongly associated with the induction of gene expression, such as PPAR*α*, the enhancement of fatty acid oxidation, and suppression of hepatic lipid accumulation. It is a noteworthy finding of the present study that the treatment with CNE markedly increased the phosphorylation of AMPK. Based on the reports of Minokoshi et al. [51], the AMPK phosphorylation by CNE may be linked to leptin secretion. There were two possibilities that CNE could directly activate AMPK or increase the secretion of leptin by inducing AMPK activation. The target molecule for CNE should be identified.

Analysis of WAT histology showed that C1 and C2 treatment decreased the number of large adipocytes and increased the number of small adipocytes (Figure 2). Since lipids that accumulate in adipose tissue are largely derived from circulating TG [52] and liver is a major target tissue for lipid and lipoprotein metabolism, CNE may be able to mobilize fat from adipose tissue by increasing lipid catabolism in liver. Based on our results, the increased fatty acid oxidation and possibly decreased TG synthesis in liver effectively regulated morphometric adipocytes.

Recently, the researchers have shown that leptin could substitute for insulin to control blood sugar fluctuations in patients with type 1 diabetes [53]. Furthermore, the researchers showed that, in a mouse model of type 1 diabetes, leptin was as effective as insulin in controlling blood sugar; nevertheless the mechanism of action is far from clear. The study demonstrates that CNE had favorable effect on leptin levels, suggesting that CNE plays a role in glucose metabolism.

Metformin has been approved since 1994 by the US Food and Drug Administration for the treatment of diabetes. Metformin decreases hepatic glucose output and increases glucose uptake in the skeletal muscle [54]. Metformin exerts an insulin-sensitizing effect through AMPK pathway [55]. In this study, Metf decreases glucose production by downregulation of PEPCK and increases glucose uptake by increasing muscular GLUT4 proteins. These results were in accordance with the results of Woods et al. [54]. Moreover, our results demonstrated that Metf increases phospho-AMPK both in liver tissue and skeletal muscle. AMPK is known to play a role in glucose and lipid metabolism. Our results indicate that Metf exerts the lipid-lowering effect through regulation of lipolysis and lipogenesis via altering the expressions of hepatic ATGL and DGAT2 in STZ induced mice. Our findings indicate that Metf not only improves lipid metabolism but also has beneficial glucose metabolism both in liver and skeletal muscle.

In conclusion, CNE increased the phosphorylation of AMPK both in skeletal muscle and liver tissue. This activation of hepatic AMPK leads to a reduction gluconeogenesis (downregulated PEPCK and G-6Pase expression), thus resulting in reduced glucose level. Moreover, CNE also increased the protein contents of muscular GLUT4 to elevate glucose uptake, thus resulting in lowering blood glucose. This activation of hepatic AMPK caused a decrease in hepatic triglyceride synthesis (downregulated DGAT2 expression), whereas an increase in fatty acid oxidation, which, in turn, contributed to the lowering of circulating triglycerides. Theoretically, activation of AMPK provides an explanation for many of the pleiotropic beneficial effects of CNE. Our findings demonstrated that CNE exerted antidiabetic and hypolipidemic properties in STZ induced diabetic mice.

## Figures and Tables

**Table 1:** Primers used in this study.

Gene	Accession numbers	Forward primer and reverse primer	PCR product (bp)	Annealing temperature (°C)
*Liver *				
DGAT2	NM_026384.3	F: AGTGGCAATGCTATCATCATCGT R: AAGGAATAAGTGGGAACCAGATCA	149	50
G6Pase	NM_008061.3	F: GAACAACTAAAGCCTCTGAAAC R: TTGCTCGATACATAAAACACTC	350	50
11*β*-HSD1	NM_008288.2	F: AAGCAGAGCAATGGCAGCAT R: GAGCAATCATAGGCTGGGTCA	300	50
PPAR*α*	NM_011144	F: ACCTCTGTTCATGTCAGACC R: ATAACCACAGACCAACCAAG	352	55
PEPCK	NM_011044.2	F: CTACAACTTCGGCAAATACC R: TCCAGATACCTGTCGATCTC	330	52
ATGL	AY894805	F: AGG ACA GCT CCA CCA ACA TC R: TGG TTC AGT AGG CCA TTC CT	165	50
GAPDH	NM_031144	F: TGTGTCCGTCGTGGATCTGA R: CCTGCTTCACCACCTTCTTGA	99	55

**Table 2:** Absolute tissue weight, weight gain over 4-week treatment (g), and blood profiles.

Parameter	CON	STZ	STZ + C1	STZ + C2	STZ + C3	STZ + Metf
			0.2^a^	0.5^a^	1.0^a^	0.3^a^
*Absolute tissue weight (g) *						
EWAT	0.235 ± 0.009	0.075 ± 0.007^###^	0.058 ± 0.010	0.061 ± 0.005	0.067 ± 0.011	0.090 ± 0.010
MWAT	0.305 ± 0.014	0.216 ± 0.011^###^	0.197 ± 0.018	0.237 ± 0.017	0.247 ± 0.015	0.250 ± 0.014
RWAT	0.024 ± 0.002	0.016 ± 0.009	0.007 ± 0.002	0.007 ± 0.003	0.005 ± 0.001	0.012 ± 0.003
Visceral fat	0.259 ± 0.010	0.091 ± 0.012^###^	0.065 ± 0.011	0.070 ± 0.007	0.072 ± 0.012	0.102 ± 0.012
BAT	0.081 ± 0.010	0.100 ± 0.049	0.056 ± 0.004	0.044 ± 0.006	0.052 ± 0.003	0.048 ± 0.003
Liver (g)	0.992 ± 0.032	1.031 ± 0.048	1.048 ± 0.031	1.063 ± 0.064	1.121 ± 0.049	1.074 ± 0.020
Spleen	0.073 ± 0.004	0.055 ± 0.003^#^	0.069 ± 0.009	0.061 ± 0.006	0.056 ± 0.002	0.055 ± 0.003
Skeletal muscle	0.270 ± 0.021	0.175 ± 0.012^###^	0.155 ± 0.014	0.147 ± 0.016	0.175 ± 0.012	0.183 ± 0.005
Weight gain (g)	0.10 ± 0.32	−0.19 ± 0.73	−0.92 ± 0.20	−1.24 ± 0.35	−0.58 ± 0.70	−0.79 ± 0.67
*Blood profiles *						
TC (mg/dL)	95.0 ± 5.9	140.7 ± 7.3^###^	160.0 ± 10.4	157.5 ± 6.2	153.3 ± 6.8	114.3 ± 7.8*
Insulin (*μ*U/mL)	10.27 ± 0.59	4.07 ± 0.38^###^	8.88 ± 0.81**	11.08 ± 1.87**	13.29 ± 0.79***	11.25 ± 1.67**

All values are means ± S.E. (*n* = 9). ^#^ *P* < 0.05, ^##^ *P* < 0.01, and ^###^ *P* < 0.001 compared with the control (CON) group; *P < 0.05, **P < 0.01, and ***P < 0.001 compared with the streptozotocin (STZ) + vehicle (distilled water) (STZ) group. C1, C2, and C3: extracts of *Clitocybe nuda.* BAT: brown adipose tissue; EWAT: epididymal white adipose tissue; RWAT: retroperitoneal white adipose tissue; MWAT: mesenteric white adipose tissue; EWAT + RWAT: visceral fat; TC: total cholesterol; TG: triglyceride.

^a^Dose (g/kg/day).

## Abbreviations

AMPK: AMP-activated protein kinase
ATGL: Adipose triglyceride lipase
BAT: Brown adipose tissue
11*β*-HSD1: 11beta hydroxysteroid dehydrogenase
CON: Control
DGAT: Acyl-coenzyme A:diacylglycerol acyltransferase
FFA: Free fatty acid
GLUT4: Glucose transporter 4
G-6Pase: Glucose-6-phosphatase
HF: High-fat control
Metf: Metformin
PEPCK: Phosphoenolpyruvate carboxykinase
PPAR*α*: Peroxisome proliferator-activated receptor *α*
PPARs: Peroxisomal proliferator-activated receptors
STZ: Streptozotocin
TC: Total cholesterol
TG: Triglyceride
WATs: White adipose tissues.
